# Challenge of ending TB in China: tuberculosis control in primary healthcare sectors under integrated TB control model–a systematic review and meta-analysis

**DOI:** 10.1186/s12889-023-16292-5

**Published:** 2024-01-11

**Authors:** Xi Chen, Jiani Zhou, Quan Yuan, Rui Zhang, Chunji Huang, Ying Li

**Affiliations:** 1 Department of Social Medicine and Health Service Management, College of Preventive Medicine, Army Medical University (Third Military Medical University), No. 30 Gaotanyan Road, Shapingba District, Chongqing, 400038 China; 2 Army Medical University (Third Military Medical University), Chongqing, China

**Keywords:** Tuberculosis, Tuberculosis control service, Primary health care sector, Systematic review

## Abstract

**Background:**

China has the third-largest burden of tuberculosis (TB) cases in the world with great challenges towards ending TB. Primary health care (PHC) sectors play a critical role in TB prevention and control in communities under the Chinese integrated TB control model. However, there is a lack of comprehensive review of research evidence on TB control in PHC sectors under the integrated TB control model in China.

**Methods:**

This review was conducted following the PRISMA guidelines. Articles published from 2012 to January 2022 were searched from four international and three Chinese databases. Studies conducted inside mainland China and relevant with TB control service in PHC sectors under the integrated model were included. After study selection, data extraction, and quality assessment, the meta-analysis was performed with RevMan using a random-effect model.When I^2^ was more than 50%, subgroup analysis was performed to explore possible reasons for heterogeneity. We also conducted a post hoc sensitivity analysis for outcomes after meta-analysis by exclusion of studies with a high risk of bias or classified as low quality.

**Results:**

Forty-three studies from 16 provinces/municipalities in China were included in this review, and most studies included were of medium quality. PHC sectors in East China delivered TB control service better overall than that in West China, especially in tracing of patients and TB case management (TCM). In meta-analyses, both the pooled arrival rate of tracing and pooled TCM rate in East China were higher than those in West China. TB patients had a low degree of willingness to receive TCM provided by healthcare workers in PHC sectors nationwide, especially among migrant TB patients. There were 9 studies reporting factors related to TB control service in PHC sectors, 6 (2 in East and 4 in West China) of which indentified several characteristics of patients as associated factors. The context of PHC sectors was demonstrated to influence delivery of TB control service in PHC sectors in 5 studies (3 in East, 1 in Middle and 1 in West China). Most studies on strategies to promoting TB control services in PHC sectors were conducted in East China and some of these studies identified several online and offline interventions and strategies improving patients’ treatment compliance [pooled OR (95% CI): 7.81 (3.08, 19.19] and awareness of TB [pooled OR (95% CI): 6.86 (2.16, 21.72)].

**Conclusion:**

It is of urgent need to improve TB control in PHC sector in China, particularly in West China. Formative and implementation research with rigorous design are necessary to develop comprehensive, context-specific, and patient-centered TB control strategies to promote ending TB in China.

**Supplementary Information:**

The online version contains supplementary material available at 10.1186/s12889-023-16292-5.

## Introduction

Before the coronavirus (COVID-19) pandemic, tuberculosis (TB) was the leading cause of death from a single infectious agent [1]. TB is caused by the bacillus *Mycobacterium tuberculosis*, and around a quarter of the world’s population is infected with M. Tuberculosis [1]. World Health Organizations (WHO) launched many strategies to promote TB control including directly observed treatment short-course (DOTS) strategy [2] and the “Ending TB Strategy” which has a blueprint of making the world free of tuberculosis by the year 2035 [3]. Although declines in TB incidence (the number of people developing TB each year) were achieved in previous years, the outbreak of the COVID-19 pandemic restricted access to TB diagnosis and treatment, resulting in an increase in TB deaths [1]. Therefore, TB is still considered as a significant challenge to public health and a threat to the global population.

China accounts for the third largest number (7.4%) of the global total estimated incident TB cases, with a high burden of multidrug-resistant TB (MDR-TB) or rifampicin-resistant TB (RR-TB) [1]. China has positively committed to eliminating TB by 2035, which means that the TB incidence rate in China needs to continuously decline and reach 33/100,000, 13/100,000, and 7/100,000 in 2025, 2030, and 2035, respectively [4]. However, TB morbidity and mortality rate have not declined as rapidly as expected in China. The incidence of TB in China only decreased from 141.83/100,000 in 1990 to 55.55/100,000 in 2019 [5, 6], and the notification rates of TB in four provinces of West China were beyond 100 cases per 100,000 population [7]. Therefore, China still faces great challenges towards ending TB by 2035.

Since the 12th Five-Year Plan of the National TB Program in 2012, most regions of China have gradually implemented the “integrated TB control model”, which involves the center for disease control and prevention (CDC), designated TB hospitals and primary health care (PHC) sectors [8, 9]. Under the integrated TB control model, PHC sectors including community health center (CHC)/station (CHS), township health center (THC) and village clinic (VC), are responsible for the following TB control service: screening and referral of TB patients and persons with presumed TB, tracing of TB cases, TB case management (TCM) and TB health education [10, 11]. Since 2015, all TB control services delivered by PHC sectors have been included in the package of national *Basic Public Health Service* (BPHS), a program to promote universal and equal basic public health services nationwide, for TB patients free of charge in China, named “the health management for patients with tuberculosis” [12, 13]. Notably, the integrated TB control model has been implemented for around ten years and many studies have reported impressive outcomes in local PHC sectors under the integrated model regarding TB prevention and control [14–16] and treatment adherence among TB patients [17–19], by adopting several main and routine indicators recommended by National Tuberculosis Information Management System, including screening rate, referral rate, tracing rate, arrival rate, TCM rate [14, 19]. However, studies also reported that TB control services in PHC sectors still faced various challenges [20, 21]. In 2021, a study by Long et al. found that healthcare workers (HCWs) in PHC sectors were not incentivized to make referrals and management for TB cases effectively under integrated TB control model [22]. Recently, another study found HCWs who delivered TB control health service in PHC sectors had heavy workload as they had to deliver several other BPHS projects due to the shortage of HCWs in PHC sectors [23]. Unfortunately, results of these studies with small samples or using only qualitative research methods were largely inconclusive and inconsistent. Inadequate TB control service in PHC sectors has potentially become a great challenge for ending TB in China [22]. Therefore, it is of urgent need to systematically understand the status of TB control service delivery in PHC sectors, the determinants of TB control service delivery, and possible responsive strategies to improve it. This understanding could inform evidence-based policy making on the improvement of TB control in PHC sectors.

To our knowledge, there is still a lack of systematic review that can provide a comprehensive analysis of research evidences on TB control in PHC sectors under the integrated TB model in China. This study aimed at critically summarizing available literature on the status of, factors related to, and strategies towards TB control services in PHC sectors of China. Findings from this systematic review could provide evidence to inform policy making on the optimization of TB control service in PHC sectors.

## Methods

### Search strategy

This review was conducted according to the standard procedures of the Cochrane Collaboration [24] and the Preferred Reporting Items for Systematic Reviews and Meta-Analyses (PRISMA) checklist (see Additional file 1) [25]. We searched for all manuscripts published in English and Chinese related to the theme of TB control in PHC sectors of China. To identify eligible studies published from 2012, when the integrated model was carried out nationwide, up to January 2022, we searched international databases involving PubMed, Cochrane, EMBASE, Web of Science and three major Chinese databases: CNKI (China National Knowledge Infrastructure), Wanfang Med Online and VIP database. We used a mixture index terms and free text to maximize retrieval of potentially relevant studies. The terms of “tuberculosis”, “TB”, “tuberculosis control”, “primary health care sector”, “community health centre”, “community health station”, “township health centre”, “village clinic”, “China” and “Chinese” were used as MeSH terms and text words. Three combinations of free texts and terms were used to maximize the retrieval of potentially relevant studies (see Additional file 2). Reference lists of identified manuscripts were hand-searched.

### Inclusion and exclusion criteria

The inclusion criteria included:

1. Type of studies: cross-sectional, case-control, and cohort and intervention studies (before and after, non-randomized trial or randomized controlled trial (RCT)).
2. Participants: TB, DR-TB, MDR-TB patients or persons with presumed TB and residents who sought care or were managed in PHC sectors, HCWs in PHC sectors, HCWs in TB designated hospitals, and leaders related to TB control from local CDC or health committees.
3. Outcome variables:

(i) Status of delivering TB control service in PHC sectors: screening rate, referral rate, tracing rate, arrival rate, TCM rate, the willingness/unwillingness of TCM, and the awareness of TB.
(ii) Factors related to delivering TB control service in PHC sectors: factors associated with screening, referral, tracing and TCM of TB patients and TB health education in PHC sectors.
(iii) Strategies towards promoting TB control service delivery in PHC sectors: any innovative or modified strategies to promote screening, referral, tracing and TCM of TB patients and TB health education in PHC sectors with outcomes including screening rate, referral rate, tracing rate, arrival rate, TCM rate, the willingness/unwillingness of TCM, the adherence to treatment, the awareness of TB, the score of support utilization (an index indicating patients’ health awareness and initiatives to seek care), and the self-management precursor score (an index indicating patients’ ability of self-management).

The exclusion criteria were: (1) articles conducted outside mainland China, such as studies focusing on Hong Kong, Macao and Taiwan were excluded; (2) articles irrelevant with TB control service in PHC sectors under the integrated model; (3) articles with incomplete data; (4) review articles; (5) articles without full texts;

If there were multiple reports of the same study, only the article with a full report was included.

### Study selection

Two reviewers (XC and YL) identified studies using the inclusion and exclusion criteria. Each reviewer screened the titles and abstracts of identified studies independently to preliminarily assess their eligibility according to the inclusion/exclusion criteria. All reviewers made the decision to include/exclude a study by discussion and consensus where there were disagreements regarding the eligibility of studies.

### Quality assessment

Two reviewers (XC and YL) independently assessed the quality of included studies. We assessed the quality of cross-sectional studies by using a checklist containing 11 items, which was recommended by the Agency for Healthcare Research and Quality (AHRQ) (see Additional file 3) [26]. An item in each study was scored ‘0’ if it was answered ‘No’ or ‘Unclear’, and ‘1’ for the answer of ‘Yes’. In the review of each study, we assigned a composite quality score as follows: low quality = 0–3; medium quality = 4–7; high quality = 8–11.

For cohort studies, we used the Newcastle-Ottawa Scale with 9 items [27]. After reviewing the quality of each included study on the basis of these criteria, we assigned quality score for each item with ‘1’ indicating the study met the criteria, or ‘0’ for the study that did not meet the criteria. Totally, study quality was assessed as follows: low quality = 0–3; medium quality = 4–6; high quality = 7–9.

For before and after studies and non-randomized trials, we used the JBI appraisal criteria for Quasi-Experimental Studies comprising 9 items [28]. For each item in quality assessment criteria, score of ‘1’ indicates the answer of ‘Yes’, score of ‘0’ indicates the answer of ‘No’ or ‘Unclear’, and ‘N/A’ meant not applicable. Total quality score of each study was assessed as follows: low quality = 0–3; medium quality = 4–6; high quality = 7–9.

In quality assessment of RCTs, we referred Cochrane Collaboration’s tool and assessed the generation of the allocation sequence, the concealment of allocation, blinding and completeness of follow-up [29, 30].

### Data extraction

Two reviewers (XC and JZ) independently read the full texts of all initially selected manuscripts and finally included eligible articles according to the inclusion/exclusion criteria. Differences were resolved by discussion and consensus among all reviewers. We established an Excel form with a reference number assigned for each included article, and then extracted following data from each study: name of first author, publication year, geographic region, and the province/municipality city where the study was carried out, type of study design, data collection methods, participants and sampling size, and the main results. Each extracted data was classified and summarized on outcome variables, and was recorded in the Excel form. Any disagreement was resolved through discussion and consensus among all reviewers.

### Data analysis

At first, we presented a qualitative synthesis aimed at summarizing, comparing and contrasting the extracted information from all included studies. Then meta-analyses were conducted through RevMan 5.2 Software [31] only if there were at least three estimates for each outcome variable, in view of potentially limited number of studies. We used random-effect model to pool odds ratios (OR) and 95% confidence intervals (CI) to calculate weight proportion for each outcome variable with the Log transformation analysis, followed by the inverse variance method. Herein, we used Log transformation analysis for outcome variable in the form of rate (rate = ln(odds) = ln(n_events_/(n_total_-n_events_)), standard error (SE) = SE (ln(odds)) = (1/n_events_+1/(n_total_-n_events_))^0.5) regardless of normal distribution of included data. After meta-analysis, each pooled OR and its 95% confidence intervals were inverted into pooled rate (= OR/(1 + OR)) and 95% confidence intervals (= limit/(1 + limit)).

Heterogeneity was evaluated using the Q test [32] and the I^2^ statistic [33]. We calculated I^2^ if P-value was ≤ 0.10, and a level of heterogeneity with I^2^ ≤ 50% was considered acceptable. When I^2^ was more than 50%, subgroup analysis based on type of intervention (offline or online) in intervention studies or geographic region of study location in other studies was performed to explore possible reasons for the heterogeneity. We assessed the heterogeneity within groups in subgroup analyses through same statistical methods which were used for heterogeneity analysis between groups as described above. After meta-analyses and subgroup analyses, we assessed the potentiol publication bias of studies in outcome variables, which contained at least five estimates, through funnel plots [31]. Furthermore, we also conducted a post hoc sensitivity analysis for outcomes after meta-analysis by excluding studies with potentially high risk of bias or classified as low quality that might introduce risk to the validity of study findings.

### Definitions

*Screening rate* refers to the proportion of persons with presumed TB who were screened by PHC sectors.

*Referral rate* refers to the proportion of TB patients/persons with presumed TB or close contacts who were referred from PHC sectors to designated hospitals.

*Tracing rate* refers to the proportion of TB patients/persons with presumed TB or close contacts who missed or delayed in arrival and were traced by PHC sectors.

*Arrival rate* refers to rate of TB patients/persons with presumed TB arriving at designated hospitals after referral or tracing.

*TCM Rate* refers to the proportion of TB patients who received case management including DOT and follow-up during the whole treatment course by PHC sectors.

*Standard TCM* refers to at least once TCM per 10 days in the intensive phase of treatment and once TCM every month during the continuous phase, which means a total of at least 10 times TCM for drug-sensitive TB patients or at least 24 times TCM for drug-resistant TB (DR-TB) patients during the treatment course [12].

## Results

### General description of studies

**Fig. 1:**
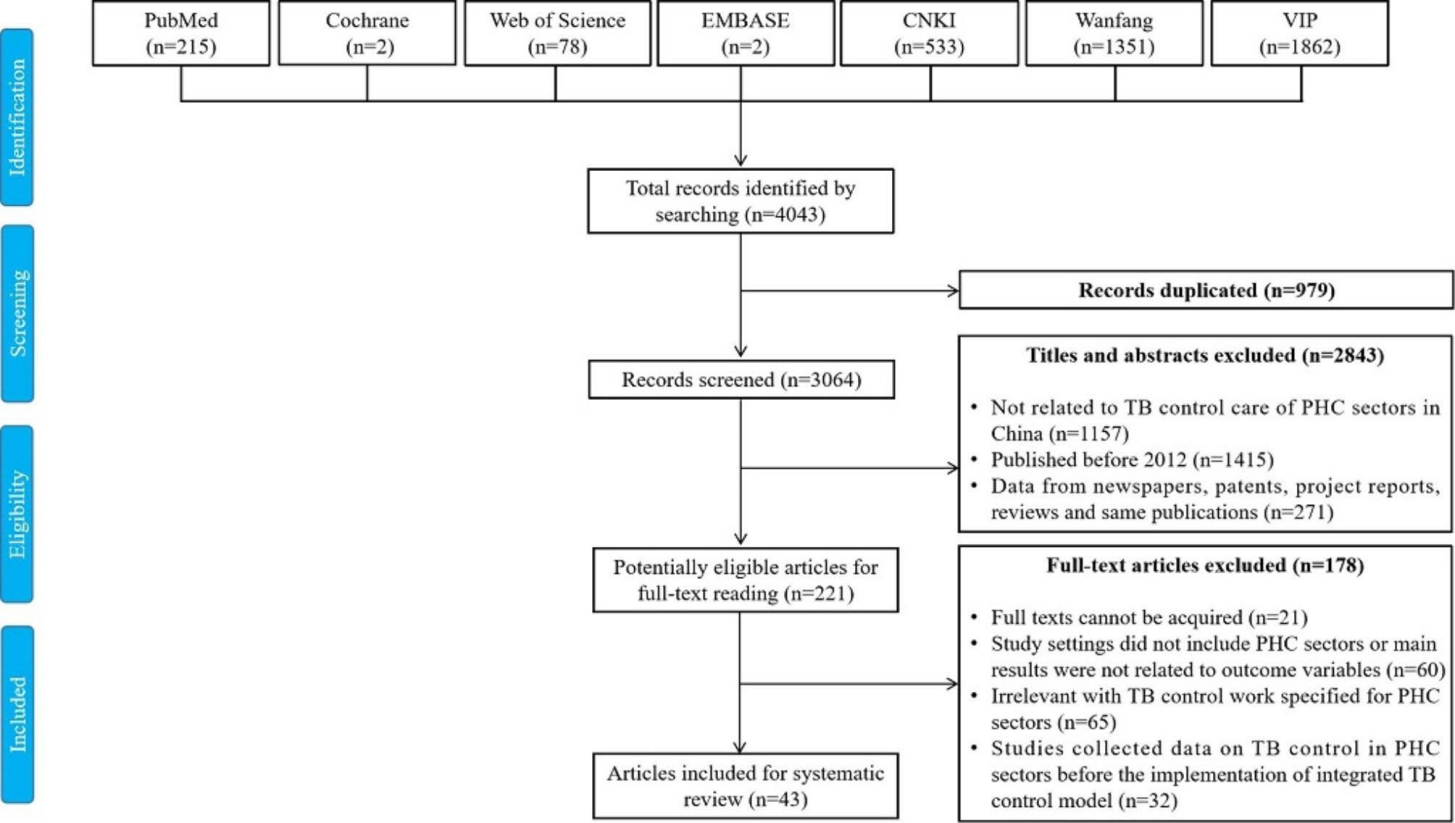
The flow diagram of the literature search process and results

Search results were illustrated in Fig. 1. We initially acquired 4,043 potentially relevant manuscripts. After excluding duplicates and reviewing titles and abstracts, we included 221 articles for full-text screening. Then twenty-one studies were excluded because the full text cannot be acquired; 60 articles did not include PHC sectors or their main results were not related to outcome variables; 65 studies were irrelevant to TB control work in PHC sectors; and 32 studies collected data on TB control in PHC sectors before the implementation of integrated TB control model (Fig. 1). Finally, 43 articles (8 in English and 35 in Chinese) from 16 provinces/municipalities in China met the inclusion criteria and were included in the review [14–19, 34–70]. The included studies comprised 25 cross-sectional, 2 cohort and 16 intervention studies (3 before and after studies, 7 non-randomized trials and 6 RCTs). A majority of the studies (28 out of 43) reported on the status of delivering TB control service in PHC sectors (Table 1), 9 articles analyzed factors related to TB control service in PHC sectors (Table 2), and 14 studies were about strategies towards improving TB control service in PHC sectors (Table 3).

**Table 1:** Studies reporting the status of delivering TB control service in PHC sectors

Author	Year	Location	Type of study	Data collection and sampling size	Main results
Jiang HX [34]	2013	East (Zhejiang Province)	CS	Review of registration data with 180 persons with presumed TB	1.Tracing rate:100%; 2.Arrival rate of tracing: 100%.
Xu WX [35]	2015	East (Zhejiang Province)	CS	Review of registration data with 7521 persons with presumed TB	Arrival rate of tracing: >4/5.
Yang SY [36]	2016	East (Shanghai Municipality)	CS	Questionnaires with 521 residents	HE: Main ways to learn TB knowledge: around 3/5 from TV vs. 1/3 from displays, leaflets or posters.
Zheng YH [17]	2016	East (Shanghai Municipality)	CS	Review of registration data with 1904 TB patients	1.Tracing rate:100%; 2.Arrival rate of tracing: 90%; 3.TCM rate: 100%; 4.Screening rate of close contacts: 100%.
Zhong T [37]	2016	East (Guangdong Province)	CS	Questionnaires with 198 TB patients	TCM: Around 1/4 patients were not followed up as required.
Liu T [38]	2016	East (Jiangsu Province)	CS	Review of outcome data with 34 TB patients	TCM: About 1/5, 1/3 and 1/3 patients accepted HCWs’ home visits, follow-up in PHC sectors, and follow-up through phone respectively and around 1/10 refused follow-up by HCWs.
Gao AH [15]	2017	East (Jiangsu Province)	CS	Review of registration data with 182 persons with presumed TB and 41 TB patients in one THC and 18 VCs	1.Referral rate: 100%; 2.Arrival rate of referral: 100%; 3.TCM rate: 100%.
Chen JH [39]	2017	East (Guangdong Province)	CS	Interviews with 30 HCWs in PHC sectors	TCM:HCWs reported the rates of DOT and follow-up were not high.
Yin J [40]	2018	East (Zhejiang Province)	Cohort	In-depth interviews with 10 TB patients	TCM: Many patients reported unwillingness to receive DOT.
Ye HM [18]	2019	East (Guangdong Province)	IS(non-randomized)	Review of registration data with 196 persons with presumed TB and 873 TB patients	1.Referral rate: close to 100%; 2.Arrival rate of referral: close to 100%; 3.TCM rate: 100%; 3.Rate of STCM: close to 100%.
Guo WR [41]	2019	East (Guangdong Province)	CS	Questionnaires with 210 migrant TB patients and 16 CHCs	1.Referral rate: < 3/5; 2. Screening rate: about 1/10.
Ou QY [42]	2019	East (Guangdong Province)	CS	Questionnaires with 72 and in-depth interviews with 6 HCWs in PHC sectors	TCM:1.Tracing rate: <4/5; 2. Over 1/10 HCWs never made the first home follow-up; Interviews: HCWs stated that some patients refused regular follow-up.
Lu QW [43]	2021	East (Shanghai Municipality)	RCT	Questionnaires with 375 students	HE: About 3/5 and 1/2 students learned TB knowledge from leaflets and posters before and after intervention, respectively.
Zhang LM [44]	2021	East (Shandong Province)	IS(non-randomized)	Review of outcome data with 320 TB patients	1.TCM rate: 100%; 2.Rate of STCM: >90%.
Liu XJ [14]	2012	Middle (Hubei Province)	IS(non-randomized)	Review of registration data with 570 persons with presumed TB	Arrival rate of tracing: about 3/4 after the implementation of the integrated model vs. 1/2 before.
Li WZ [16]	2013	Middle (Hubei Province)	CS	Questionnaires with 102 TB patients	Arrival rates of referral and tracing: both around 90%.
Wu TY [45]	2014	West (Guangxi Province)	CS	In-depth interviews with 20 HCWs in PHC sectors	TCM: Some migrant TB patients refused HCWs’ home visits, and TCM for migrant TB patients were difficult.
Li Y [46]	2014	West (Chongqing Municipality)	CS	Questionnaires with 513 TB patients	HE: <1/5 TB patients accessed to TB health education from PHC sectors.
Huang F [47]	2016	West (Ningxia Autonomous Region)	IS(non-randomized)	Review of registration data with 1185 persons with presumed TB	Arrival rate of referral: close to 100%.
Chen W [48]	2016	West (Guizhou Province)	CS	Questionnaires with 10,237 residents	HE: 1.Vulnerable groups (women, people with less education, older people or underemployed adults) had low access to TB health education; 2.Main ways to learn TB knowledge: TV (about 1/3) vs. relatives and friends (about 3/10) vs. leaflets (about 3/10).
Lin B [49]	2017	West (Xinjiang Autonomous Region)	CS	Semi-structured interviews with 93 HCWs in PHC sectors	TCM, HE: Few patients did not received TB health education and were unwilling to receive TCM.
Mi YS [50]	2018	West (Xinjiang Autonomous Region)	CS	Questionnaires with 171 TB patients	HE: Main ways to learn TB knowledge: propaganda(about 3/5) and leaflets and posters (about 1/2).
Zhang HW [19]	2019	West (Shaanxi Province)	CS	Review of registration data with 118,266 TB patients	1.Referral rate: about 4/5; 2.Arrival rate of referral: about 2/3; 3.TCM rate: close to 100%.
Li JH [51]	2019	West (Gansu Province)	IS(non-randomized)	Review of registration data with 1336 TB patients	TCM rate: close to 100%.
Pu J [52]	2019	West (Guizhou Province)	CS	Questionnaires with 638 TB patients	TCM: 1.Rate of TCM: <2/5; 2.About 2/5 TB patients were unwilling to receive TCM.
Ji XF [53]	2020	West (Gansu Province)	CS	Review of registration data with 43 TB patients	TCM rate: close to 100%.
He YY [54]	2020	West (Guizhou Province)	CS	Questionnaires with 117 TB patients	TCM rate: close to 100%.
Xing W [55]	2021	West (Chongqing Municipality)	CS	Questionnaires with 132 and in-depth interviews with 2 MDR-TB patients	TCM: 1.TCM rate: about 3/5; 2. Around 1/3 patients received TCM via phone and home visits vs. around 1/4 via visits to the clinic vs. 1/10 via home visits; Interviews: Some patients reported receiving TCM only via phone, no access to TB health education from PHC sectors and one reported unwillingness to receive TCM.

CS, cross-sectional study; RCT, randomized controlled trial; IS; intervention study; CHC, community health center; THC, township health center; VC, village clinic; HCWs, healthcare workers; TB, tuberculosis; DOT, directly observed treatment; HE, health education; TCM, TB case management; STCM, standard TCM

**Table 2:** Studies analyzing factors related to TB control service in PHC sectors

Author	Year	Location	Type of study	Data collection and sampling size	Associated factors
Zhong T [37]	2016	East (Guangdong Province)	CS	Questionnaires with 198 TB patients	Patients: low health awareness, changes in work.
Yin J [40]	2018	East (Zhejiang Province)	Cohort	In-depth interviews with 10 TB patients and 10 HCWs in PHC sectors	Patients: fear of privacy disclosure related to TB; HCWs: afraid of being infected, shortage of human resources;.
Ou QY [42]	2019	East (Guangdong Province)	CS	In-depth interviews with 6 HCWs in PHC sectors	HCWs: shortage of human resources.
Ou QY [56]	2019	East (Guangdong Province)	CS	In-depth interviews with 10 key informants	Health system: unclear diversion of labor and cooperation mechanism between general hospitals and PHC sectors.
Ming H [57]	2019	Middle (Hunan Province)	CS	In-depth interviews with 72 key informants	Health system: poor coordination and cooperation among CDCs, PHC sectors and designated medical institutions restricted the efficiency and quality of TB control.
Wu TY [45]	2014	West (Guangxi Province)	CS	In-depth interviews with 20 HCWs in PHC sectors	Patients: fear of privacy disclosure related to TB; HCWs: shortage of human resources.
Lin B [49]	2017	West (Xinjiang Autonomous Region)	CS	Semi-structured interviews with 93 HCWs in PHC sectors	Patients: being afraid of being discriminated against.
Pu J [52]	2019	West (Guizhou Province)	CS	Questionnaires with 638 TB patients	Patients: social stigma, low health awareness, with junior school education (OR = 0.54), living in an area with middle (OR = 0.17) and high (OR = 0.25) TB burden.
Xing W [55]	2021	West (Chongqing Municipality)	CS	Questionnaires with 132 MDR-TB patients/ in-depth interviews with 2 HCWs in PHC sectors	Patients: female (OR = 0.26), low health awareness (OR = 0.14), and social stigma associated with TB.

CS, cross-sectional study; HCWs, healthcare workers; TB, tuberculosis

**Table 3:** Studies about strategy to promoting TB control service in PHC sectors

Author	Year	Location	Type of study	Data collection and sampling size	Intervention	Main results
Ren PP [58]	2015	East (Zhejiang Province)	BAS	Questionnaires with 292 residents	Holding lectures and setting up special exhibits on TB	Awareness of TB: from about 30% to close to 90%.
Zhou QM [59]	2015	East (Zhejiang Province)	IS(non-randomized)	Questionnaires and review of registration data with 357 TB patients	HE with QQ program and WeChat	1.Awareness of TB: from about 3/4 to close to 100%; 2.Rate of adherence to treatment: from about 90% to close to 100%.
Yao CB [60]	2016	East (Zhejiang Province)	BAS	Questionnaires with 1168 residents	HE by setting up teams for education and special exhibits on TB, holding lectures and playing multimedia videos	Awareness of TB: from about 3/4 to around 3/5.
Wang N [61]	2019	East (Jiangsu Province)	Cohort	Questionnaires with 9 HCWs Review of registration data with 231 TB patients	Electronic medication monitor for TCM	1.Rate of adherence to treatment: close to 100%; 2. Around 1/10 patients were switched to DOT due to poor adherence.
Tang SP [62]	2020	East (Shandong Province)	RCT	Scales with 66 TB patients	Standard health education, medication guidance, psychotherapy and diet management vs. medication guidance and health education	Rate of adherence to treatment: 100% vs. 90%.
Li XF [63]	2020	East (Guangdong Province)	IS(non-randomized)	Review of registration data with 23,619 TB patients and persons with presumed TB	WeChat for referral and follow-up	Close to 100% patients were adherent to follow-up.
Lu QW [43]	2021	East (Shanghai Municipality)	RCT	Questionnaires with 375 students	HE with WeChat platform vs. distribution of HE manuals	1.Awareness of TB: about 2/3 vs. 1/2; 2. The rate of correct behavior: about 1/4 vs. 1/10.
Yang WJ [64]	2021	East (Beijing Municipality)	RCT	Review of outcome data with 77 TB patients	Routine whole-course management combined with supervision in community vs. routine whole-course management	Rate of adherence to treatment: >90% vs. about 3/4.
Wang JJ [65]	2021	East (Beijing Municipality)	CS	Review of outcome data with TB patients	WeChat platform for TCM	Rate of adherence to treatment: improved by 13%.
Shi YY [66]	2021	East (Shanghai Municipality)	CS	In-depth interviews with 12 HCWs in PHC sectors and 11 TB patients	Mobile APP for TCM	1. Patients frequently used modules of “medication record”, “return visit record”, and “sputum examination record” and had a high utilization rate of medication record module; 2.Most HCWs and patients believed that training on the use was needed and the elderly had difficulties in using APP.
Huang MR [67]	2021	East (Guangdong Province)	RCT	Questionnaires with 802 students	HE with application of Internet platform vs. face-to-face education and distribution of leaflets and posters	1.Average awareness of TB: from about 2/3 to close to 90%; 2. The rate of correct behavior: from about 70% to around 85%.
Li XH [68]	2018	Middle (Hubei Province)	RCT	Scales with 201 TB patients	HE, psychotherapy and support interventions vs. HE	1.The score of support utilisation increased more obviously (P < 0.05); 2.Many patients participated in social activities, sought and used help from others and were willing to disclose their disease in intervention group.
Chen Y [69]	2020	Middle (Hubei Province)	RCT	Scales with 244 TB patients	HE, psychotherapy and self-management intervention vs. HE	Self-management precursor scores in self-efficacy increased by 3.62 (P < 0.001).
Zhou JH [70]	2016	West (Sichuan Province)	BAS	Questionnaires with 1382 residents	HE by setting up teams for education and special exhibits on TB, holding lectures and playing multimedia videos	Awareness of TB: from about 1/3 to close to 100%.

BAS: before and after study; CS: cross-sectional study; RCT: randomized controlled trial; IS: intervention study; HCWs: healthcare workers; TB: tuberculosis; DOT: directly observed treatment; HE: health education; TCM: TB case management

The quality assessment of studies (see Additional file 3) demonstrated that five cross-sectional studies [45, 46, 48, 52, 55] had a high quality score beyond 8. Most of (15/25) cross-sectional studies [16, 17, 19, 34–37, 39, 41, 42, 49, 50, 54, 57, 66] were of middle quality and five [15, 38, 53, 56, 65] were considered as low quality. One cohort study [57] had a high quality score of 8, and the other [40] had a medium quality score of 6. All before and after studies [58, 65, 70] were considered as medium quality and did not describe reliable methods measuring outcomes. Among non-randomized trials included, only one trial [47] was of high quality, and six trials [14, 18, 44, 51, 59, 63] were classified as medium quality without reliable methods of outcomes measurement and complete follow-up. For RCTs, there were only two trials [43, 64] reporting the adequate generation of allocation sequence, and other four trials [62, 67–69] did not mention information on generation of allocation sequence. All RCTs had unclear description of concealment of allocation, and the completeness of follow-up in all RCTs was adequate. The outcome assessment was blind in only one trial [69] and other five trials [43, 62, 64, 67, 68] did not provided description of blinding to assessors.

### The status of delivering TB control service in PHC sectors in China

Twenty-eight studies (14 in East, 2 in Middle and 12 in West China) reported TB control service delivery in PHC sectors in China, focusing on the status of screening, the referral and tracing of TB patients/persons with presumed TB, TCM and TB health education.

### Screening, referral and tracing of TB patients and persons with presumed TB

In East China, studies on the status of TB control service in PHC sectors were mainly (13/14) conducted in 4 provinces including Zhejiang, Jiangsu, Shanghai and Guangzhou Province [17, 19, 20, 34–43]. Results of studies from Zhejiang Province and Shanghai Municipality consistently reported 100% tracing rate at the early stage of the integrated model [17, 34]. About 90% of TB patients/persons with presumed TB traced by PHC sectors in Zhejiang Province and Shanghai Municipality arrived at TB designated hospitals [17, 34, 35]. PHC sectors also delivered effective referral [15, 18] for TB patients/persons with presumed TB under the integrated TB control model in East China. Moreover, the screening rate of close contacts reached 100% in Shanghai Municipality [17], and a 100% successful referral rate of TB patients/persons with presumed TB to TB designated hospitals was reported in Jiangsu Province [15]. However, TB control service in PHC sectors in Guangdong Province faced challenges among migrant TB patients with lower screening rate and referral rate [41] and delivered poor tracing to TB patients [42].

Only two studies reported the referral of TB patients and persons with presumed TB by PHC sectors in West China. One study in Ningxia Autonomous Region reported about 100% arrival rate of persons with presumed TB referred by local PHC sectors [47], but that in Shaanxi Province was only about 2/3 [19].

**Fig. 2:**
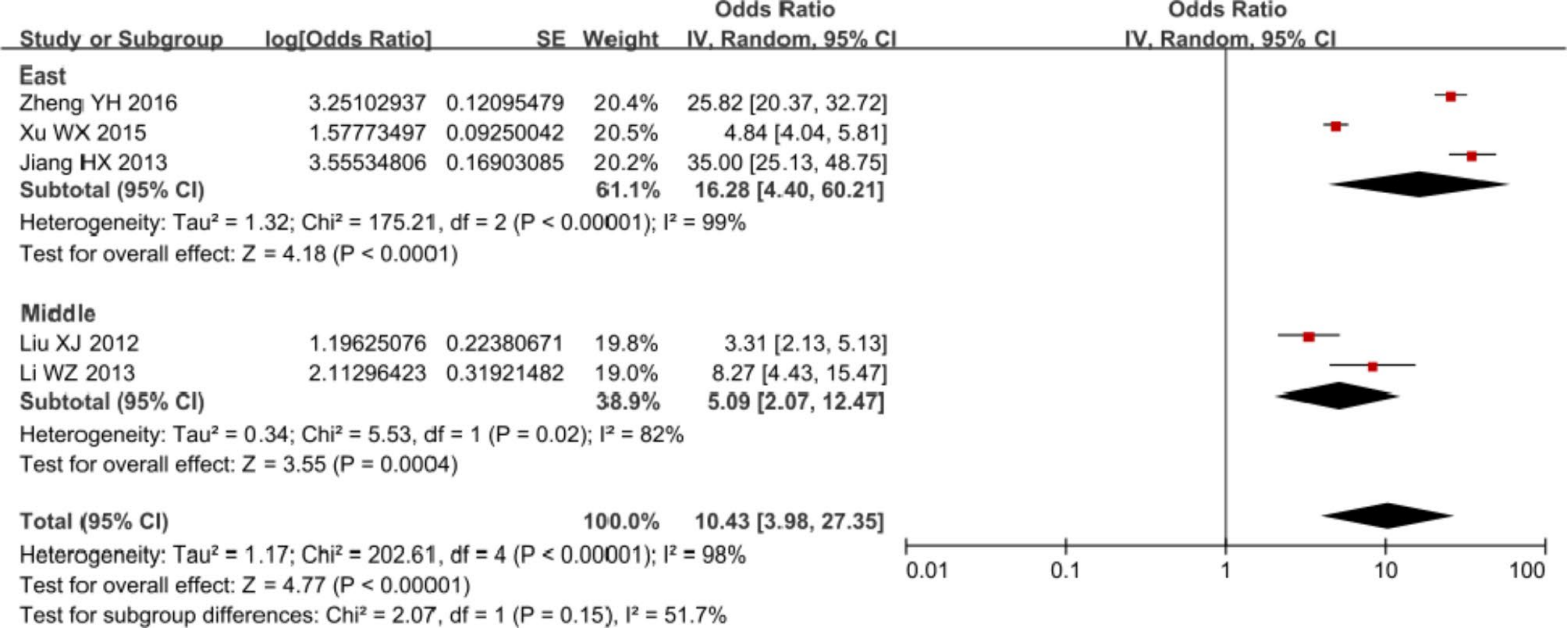
Forest plot of arrival rate of tracing in meta-analysis and subgroup analysis

**Fig. 3:**
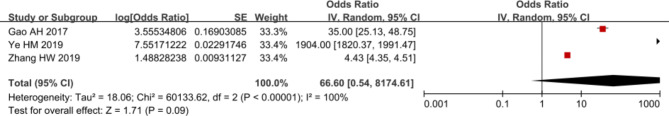
Forest plot of referral rate in meta-analysis

**Fig. 4:**
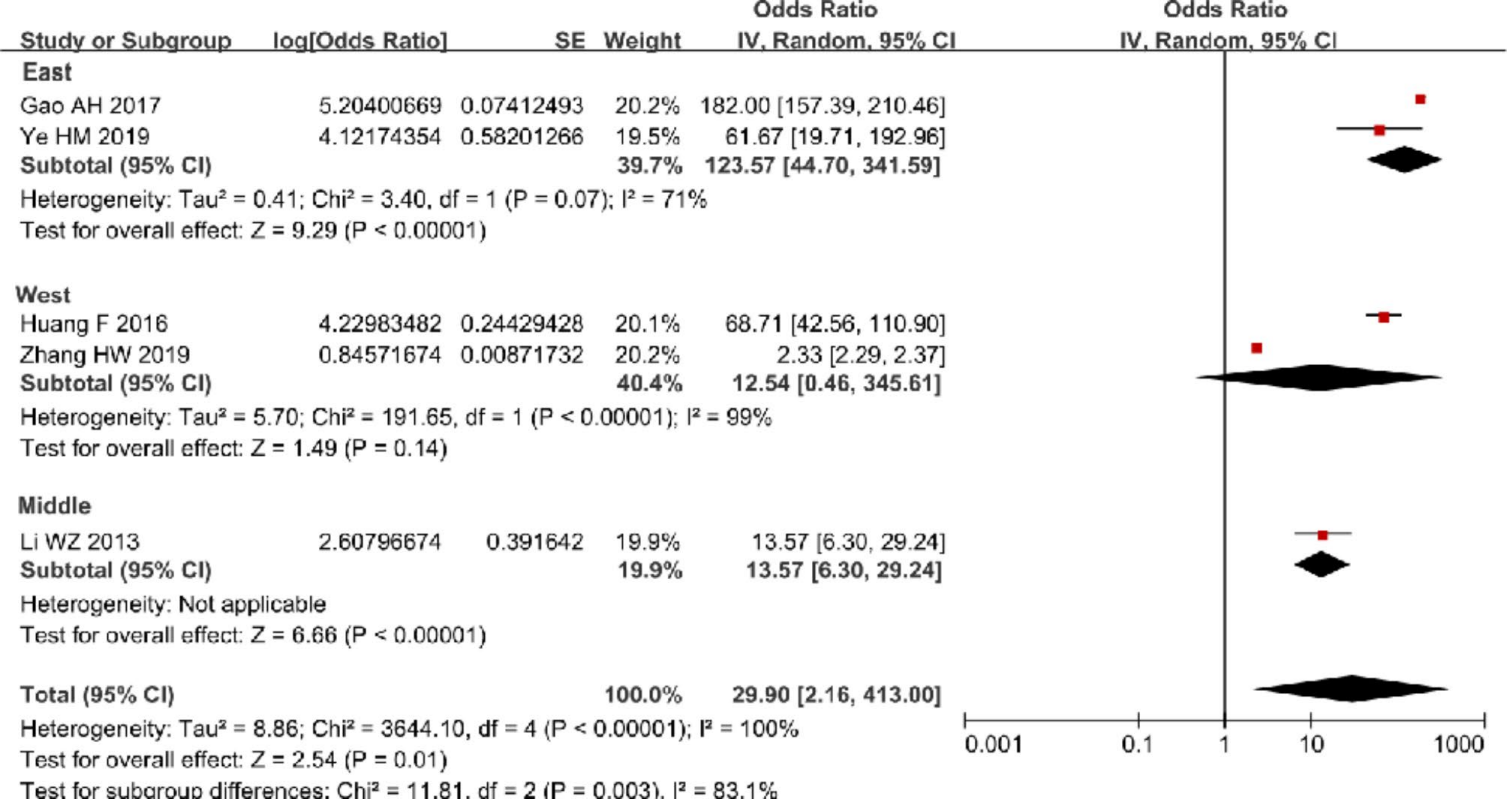
Forest plot of arrival rate of referral in meta-analysis and subgroup analysis

Results of meta-analysis showed that the pooled arrival rate of tracing was 91.25% (after transformation) (95% CI: 79.90 to 96.47%) with a high level of heterogeneity (I^2^ = 98%) (Fig. 2). Subgroup analysis suggested the pooled arrival rate of tracing in East China was 94.21% (95% CI: 81.48 to 98.37%), which was relatively higher than that in West China [83.58% (95% CI: 67.43 to 92.58%)]. The pooled referral rate was 98.52% (95% CI: 35.06 to 99.99%) with no statistical significance and great heterogeneity (I^2^ = 100%) (Fig. 3). Figure 4 indicated that the pooled arrival rate of referral was 96.76% (95% CI: 68.35 to 99.76%) and the heterogeneity was significant (I^2^ = 100%, P < 0.1). Furthermore, funnel plots of studies in both arrival rate of tracing and arrival rate of referral were almost symmetric, indicating low risks of publication bias of these studies (Additional file 4). Due to consistent classification of medium quality of all included studies for arrival rate of tracing and result with no statistical significance in all included studies for referral rate, we only conducted post hoc sensitivity analysis for arrival rate of referral after excluding one study [15] that was classified as low quality. The exclusion of this study did not reduced the heterogeneity evidently (I^2^ from 100 to 99%) and the pooled arrival rate of referral was 94.93% (95% CI: 68.94 to 99.37%) after post hoc sensitivity analysis (Additional file 5).

#### TCM

Over the past decade, research results were inconsistent on TCM service delivered in PHC sectors of China. In East China, studies in Shanghai Municipality and Jiangsu Province reported 100% TCM rates among TB patients in PHC sectors at the early stage of integrated model [15, 17]. Similarly, a study reported a 100% TCM rate in PHC sectors in Guangdong Province, East China [18]. However, other studies showed that the DOT and follow-up services for TB cases in PHC sectors were insufficient in Guangdong Province [37, 39, 42]. Notably, in recent years, two provinces in East China (Guangdong Province in 2017 and Shandong Province in 2019) achieved high rates of standard TCM for patients (close to 100% and over 90% respectively) [18, 44].

In West China, studies on TCM delivery in PHC sectors were conducted after 2019 and focused on only 4 provinces. Since the health management for TB patients was included in the BPHS in 2015, three provinces (Gansu, Guizhou and Shaanxi) made extensive coverage of TCM for TB patients, with TCM rates in PHC sectors increasing to nearly 100% under the integrated TB control model (the national standard is 90%) [19, 51, 53, 54]. However, individual studies in Guizhou Province and Chongqing Municipality observed insufficient TCM service delivery in PHC sectors of West China [52, 55] with a particular low TCM rate among MDR-TB patients (about 3/5) [55]. Moreover, it was highlighted the low quality of TCM service provided by HCWs in PHC sectors of West China [55]. Besides, in the past decade, some PHC sectors reported low acceptance of patients to TCM in both East and West China [38, 40, 42, 45, 49, 52, 55].

**Fig. 5:**
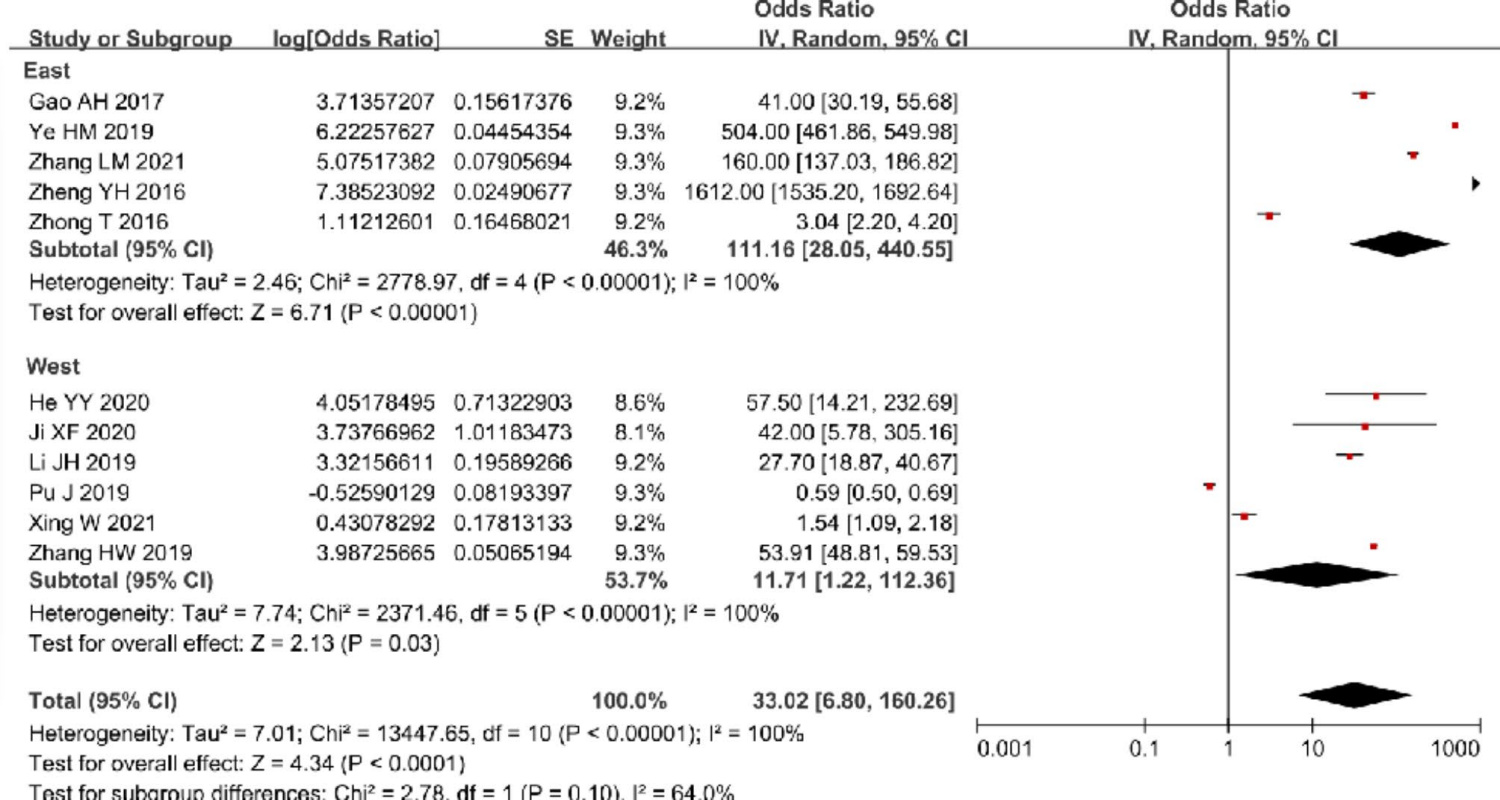
Forest plot of TCM rate in meta-analysis and subgroup analysis

In the meta-analysis, results suggested that the pooled TCM rate in 11 studies was 97.06% (95% CI: 87.18 to 99.38%) with significant heterogeneity (I^2^ = 100%) (Fig. 5). Subgroup analysis showed that the pooled TCM rate in East China was [99.11% (95% CI: 96.58 to 99.77%)] relatively higher than that in West China [92.13% (95% CI: 54.95 to 99.11%)]. Funnel plot of studies in TCM rate was almost symmetric, which indicated that the risks of publication bias of these studies were low (Additional file 4). In the post hoc sensitivity analysis for TCM rates, two studies [15, 53] with low quality were excluded, and the result indicated no reduction of the heterogeneity (I^2^ = 100%) with a pooled TCM rate of 96.92% (95% CI: 84.54 to 99.45%) (Additional file 5).

#### TB health education

Studies showed that HCWs in PHC sectors mainly relied on traditional approaches such as printed publicity materials to carry out health education over the past decade [36, 43, 48, 50]. However, individual studies in Xinjiang Autonomous Region and Chongqing Municipality in West China found that some patients did not receive TB control-related health education from PHC sectors [46, 49, 55]. A study in Guizhou Province reported low accessibility to TB health education services from PHC sectors among vulnerable TB patients, including women, people with little education, elderly or underemployed adults [48].

### Factors related to TB control services in PHC sectors

Nine studies reported factors influencing TB control in PHC sectors under the integrated TB control model (Table 2). Most of these studies (6/9) reported demographic and social factors of TB patients. For example, female patients [55] and patients with low health awareness [37, 52, 55] were less likely to receive TCM delivered by PHC sectors. Quantitative results from one study demonstrated that TB patients who lived in areas with middle and high TB burden [52] and those with junior high school education [52] were more likely to receive TCM from PHC sectors. Another study found that change in work place was associated with lower likelihood of patients’ adherence to TCM [37]. Notably, most (5/6) studies indicated that stigma related to TB was an important factor associated with TB patients’ unwillingness to receive TCM from PHC sectors, particularly in West China [40, 45, 49, 52, 55].

Few studies analyzed barriers from PHC sectors and multi-sector cooperation on TB control in PHC sectors (Table 2). Three qualitative studies reported that the shortage of human resources in PHC sectors was a barrier to TCM delivered by HCWs [40, 42, 45]. Individual studies reported the worrying about infection of TB [40], the inadequate support from CDC and TB designated hospitals [56, 57] were barriers to deliver TB control health services in PHC sectors.

### Strategies towards promoting TB control services in PHC sectors

**Fig. 6:**
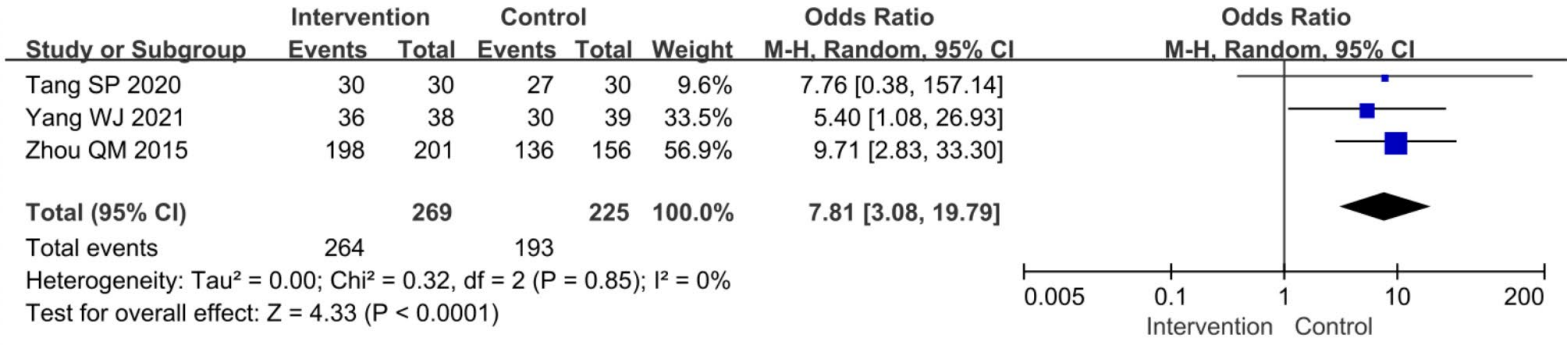
Forest plot of rate of adherence to treatment after intervention

Over the past decade, there were 14 studies about strategies to improve TB control in PHC sectors. Most of these studies (11/14) were conducted in East China and only reported outcomes collected in recent years. Nearly half (6/14) explored innovative approaches of TCM in East China (Table 3). Compared with traditional TCM approaches, both comprehensive TCM approaches integrating multiple measures [62, 64] and apps containing QQ program and WeChat [59] could consistently better improve patients’ treatment compliance [pooled OR (95% CI): 7.81 (3.08, 19.19), I^2^ = 0%] (Fig. 6). Furthermore, some studies explored the innovative technologies for TCM, such as WeChat [63, 65], electronic medication monitoring [61] and other mobile applications [66], which were effective to promote some TB patients’ adherence to treatment and follow-up examinations. However, the limitations of those technologies were obvious, for example, specific training was required and elderly people had difficulty in using them [66], which resulted in lower coverage [61, 66].

**Fig. 7:**
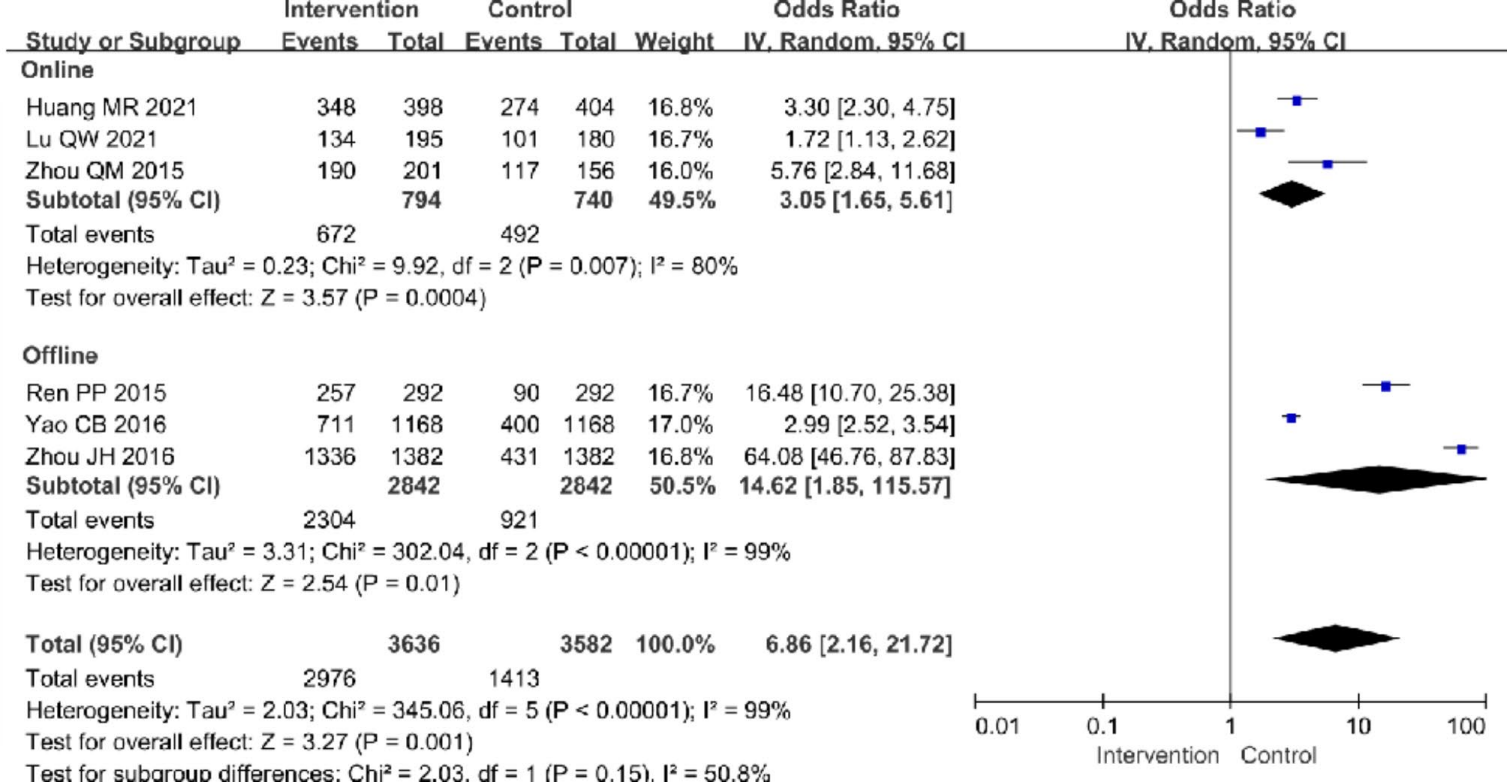
Forest plot of awareness of TB in meta-analysis and subgroup analysis

Some studies explored the innovative health education approaches and technologies for TB patients. Compared with traditional and singular health education approaches, comprehensive health education interventions could better improve TB patients’ health awareness, support seeking [68] and their efficiency of self-administration [69]. Additionally, both offline comprehensive health educational interventions, such as establishing teams consisting of HCWs in PHC sectors and TB control specialists for health education [60, 70], holding lectures [58, 60, 70], setting up special exhibits on TB [58, 60, 70], and playing multimedia videos [60, 70], and online information technologies including the Internet platform [67], QQ program [59] and WeChat [43, 59] could better improve residents’ as well as TB patients’ awareness of TB [pooled OR (95% CI): 6.86 (2.16, 21.72), I^2^ = 99%] (Fig. 7). Moreover, the result of funnel plot showed that intervention studies in awareness of TB were at low risks of publication bias (Additional file 4). The post hoc sensitivity analysis for awareness of TB after intervention explored the effect of excluding one study [67] that was classified as potentially high risk of bias. There was no change in heterogeneity (I^2^ = 99%) after the exclusion of this study and residual interventions were still effective in improving awareness of TB [pooled OR (95% CI): 7.94 (1.94, 32.56)] (Additional file 5).

## Discussion

In 2012, the implementation of the integrated TB control model was regarded as an important milestone and reform for TB control in China. Since then, many districts/counties in China had gradually established the integrated TB control model. As the front-line forces of primary health care and health promotion, PHC sector is a critical component of the integrated TB control model. It provides primary TB control services for patients in the community, which plays a key role in TB prevention and control for whole community and country [8, 9]. There were many studies on TB control in PHC sectors of China, but their focuses and results were inconsistent. Therefore, it is necessary to conduct a systematic review to understand the status of TB control service in PHC sectors in China and to identify the existing problems and associated factors, which can further provide evidences for policy-makers to take corresponding measures to improve TB control in PHC sectors.

In this article, we reviewed studies about the status of delivering TB control service in PHC sectors in China from the perspective of geographic region of study location since development of PHC sectors in China varied from East China to West China [71]. Evidences indicated that, at the early stage of the integrated model, only Zhejiang Province and Shanghai Municipality from East China provided better TB control service in PHC sectors. Later, PHC sectors in East China delivered better TB control service, including tracing and TCM of TB patients with relatively higher pooled rates in meta-analysis, overall than that in West China, as PHC sectors developed much better in East China than West China and had better capacity to provide TB control service, resulting in TB patients’ better access to TB control healthcare [72]. Notably, delivery of TB control service related to the screening, referral, and TCM among migrant TB patients was still a big challenge in PHC sectors of individual province in East China, like Guangzhou Province [37, 39, 41, 42, 73], and West China [52, 55]. Furthermore, according to the existing evidence, studies in West China mainly focused on Chongqing municipality, Guizhou and Gansu province, and TCM and TB health education in PHC sectors still need to be largely promoted, especially for MDR-TB patients and migrant TB patients in these regions. However, very few studies reported the status of TB control service in PHC sectors in other regions with high TB burden in West China, such as Xinjiang with the highest TB incidence and Tibet with the second-highest TB incidence in China [6]. What’s more, few studies investigated the screening and tracing of TB patients in PHC sectors of West China. As one of the key BPHS programmes, TCM is crucial for improving patients’ treatment adherence and treatment success rate [12, 23], nevertheless, this review disclosed that there was low acceptance to TCM provided by HCWs in PHC sectors among TB patients around the whole China. Besides, most existing studies collected data from either patients or HCWs, and only very few studies investigated the status and barriers from both TB patients and HCWs.

Current studies identified that characteristics of patients and context of PHC sectors were associated with the status of TB control service in PHC sectors. At the level of patients, firstly, many studies reported gender, residence, education, and busy work were associated with patients’ compliance to TCM. Secondly, evidences from existing studies also identified TB-related knowledge and awareness among TB patients directly affected TB patients’ willingness of accepting TCM from PHC sectors. And TB knowledge and awareness among TB patients and community residents in China were far lower than the requirement by the national TB control programme [36, 43, 48, 50]. Community residents’ lower TB-related knowledge was also associated with their stigma related to TB [74, 75], which hindered TB patients to accept TCM from PHC sectors. Interestingly, one study included in this review indicated that patients with lower-level education were more acceptable to TCM [52]. The underlying causes of distinct willingness to TCM between patients with health awareness and those with low education are worthy of further study. Moreover, other possible reasons for low acceptance to TCM may be the non-patient-centered TCM manner [52], patients’ lower trust in HCWs in PHC sectors [76], or patients’ poor awareness of TCM [55]. Thirdly, Chinese TB control program provides free treatment for TB patients, including free anti-TB drugs, 2 chest radiographs and 4 sputum smear tests, but TB patients still need to pay for drugs in terms of side effects and other examinations, transportation and accommodation [77, 78], which are much higher than the exempt medical expenditures and more likely to result in catastrophic expenditures for TB patients, especially for MDR-TB patients [55] and those in remote areas [77]. We did not identify the study that indicated the patients’ economic burden as a factor associated with their receiving of TB control service from PHC sectors. Furthermore, as a global crisis in recent years, COVID-19 pandemic heavily disrupted biomedical care for TB-affected households [79] and it was reported to cause delay of patients in seeking diagnosis in a city of China [80]. However, the impact of COVID-19 itself and its negative economic consequences on patients’ acceptance to TB control service delivered by PHC sectors in China had not been reported.

At the level of PHC sector, HCWs in PHC sectors were the main providers of TB control service in PHC. Chinese central and local governments paid high attention to develop PHC sectors and invested a lot of resources into PHC sectors since the implementation of Chinese new healthcare reform plan in 2009 [81]. However, various studies reported the shortage of HCWs in PHC sectors to deliver BPHS [20, 82] and difficulties in retaining HCWs [56, 57]. In most PHC sectors, one HCW usually needs to undertake 2–3 categories of BPHS, which results in a lack of HCWs dedicatedly delivering TB control service full-time [56]. Notably, the shortage of HCWs in PHC sectors responsible for TB control might be unprecedentedly obvious during COVID-19 pandemic with which substantial official and social resources were needed to cope [83]. Moreover, compared with other BPHS program, TB control service in PHC sectors largely increases workload for HCWs [42, 45, 53], as more time is needed to manage TB patients during the long treatment period of TB. The risk of infection from TB has also led to concerns among HCWs, and their economic incentives to TB control service remain limited in most PHC sectors [23]. This review highlighted that the shortage of HCWs dedicated to TB control service delivery and HCWs’ worry of infection were significant barriers to TB control in PHC sectors. Current evidence also disclosed the lack of HCWs with sufficient TB control-related knowledge/skills and screening equipment for patients in PHC sectors of resource-limited regions, particularly in West China [84], which were recently identified as barriers to TB control in PHC sectors [23]. Furthermore, although the National TB Program (NTP) in China emphasized wide multidisciplinary and multisectoral collaboration in TB control across the country [4], available evidences indicated that the current inadequate multisectoral collaboration between TB control system and other sectors, or cooperation within the integrated TB control system, affected TB control in PHC sectors.

In response to barriers to TB control service in PHC sectors, research on interventions/strategies is necessary. WHO encourages the development of innovative people-centered TB care strategies to improve health outcomes [85, 86]. NTP in China also strengthens research on strategies and measures for TB control and prevention [87]. The updated Technical Specifications for Tuberculosis Prevention and Control in China advocates for the application of innovative technologies, such as electronic reminder and WeChat, as the supplement for traditional DOT by HCWs in PHC sectors [43, 88, 89]. This review showed that existing studies identified the effectiveness and efficacy of several offline and online interventions/strategies on TCM and TB health education in PHC sectors. However, the effective implementations of those interventions/strategies in the real-world PHC sectors in different regions require adaptation to the local PHC sector context and TB patients because programs must be carried out in a context-specific and patient-sensitive manner [52, 83] through implementation research [90, 91].

### Strengths and limitations

This systematic review focused on the PHC sectors, which are the critical front-line forces of TB control service, and comprehensively identified and analyzed relevant studies after the implementation of integrated TB control model by narrative synthesis and meta-analysis. By specifically focusing on the standard work contents, including the screening and referral of TB patients and persons with presumed TB, tracing of TB cases, TCM and TB health education, we comprehensively summarized existing evidence on the status of, factors related to, and strategies on delivery of TB control service in PHC sectors of different regions over the past 10 years. With objective evidences about progress and barriers to TB control service in PHC sectors, findings of this study provide targets for future optimization of TB control in China.

However, this review has some limitations. Firstly, the studies included were based on published paper and we identified eligible studies and collected appropriate information as comprehensively as possible, but most included studies were of medium quality, and few were of low quality, which could potentially affect the credibility of findings of this review. Ambiguity in results and distinctly different study sizes of participants among some studies were also possible reasons contributing to the great heterogeneity, which potentially indicated the representativeness of existing studies needed to be improved. In this review, we primarily explored to obtain pooled results of outcome variables in meta-analyses even though there were limited studies with same design. The pooled results were objective but the heterogeneity was high and the extent of evidences from different types of studies varied. We tried to address the heterogeneity for each indicator by conducting subgroup analysis with the same statistical methods and a post hoc sensitivity analysis through exclusion of low-quality studies, nevertheless, the overall heterogeneity and pooled effect of our results did not change significantly and it indeed should be prudent in making conclusion, indicating the urgent need for more researches with more rigorous design to prove these results. Secondly, the number of included studies for each variable outcome was limited and there were high levels of heterogeneity of pooled results in most outcome variables included into meta-analysis. Results of subgroup-analysis suggested that geographic region of study location might be related to heterogeneity of pooled arrival rate of tracing and arrival rate of referral, and type of intervention possibly interacted with pooled awareness of TB in intervention studies, however, partial information made it impossible to conduct subgroup-analysis of other data like demographic characteristics. Thirdly, since the development of PHC sectors, socio-economy, TB burden and patients’ characteristics varied in East, Middle and West China, therefore, the findings of studies included in this review which were mainly conducted in East China could not represent those in Middle and West China. Finally, we failed to identify grey literature, which might cause the miss of some studies. The findings of this review need to be further extrapolated considering these limitations.

### Implications

As the findings of this review showed, there are more studies on TB control in PHC sectors in East China with better socio-economy and lower TB burden. To achieve the aim of Ending TB, China needs to put more efforts into research, and it is deserved to conduct more studies with better designs to investigate the status of and barriers to TB control service in PHC sectors in West China with high TB burden, which provide evidence for developing more feasible interventions.

Currently, interventions/strategies to improve TCM and TB health education in PHC sectors are the only ones being developed, leaving other barriers to delivering TB control service in PHC sectors unaddressed. Based on the practical robust implementation and sustainability model (PRISM), which is widely used as a theoretical framework in implementation research [92], successfully implementing TB control in PHC sectors is related to the intervention design (patient-centered manner, funding for health education, e.g.), recipients (patients’ economic condition, professional HCWs, incentives, e.g.), external environment (health insurance, multisectoral coordination, e.g.), and implementation and sustainability infrastructure (transportation tools, equipment, e.g.).To promote TB control in PHC sectors, comprehensive interventions/strategies based on systematic understanding of barriers to TB control in local PHC sectors should be developed through theory-based formative research. In response to the call of Ending TB strategy proposed by WHO and development of integrated TB control model in China, the design of these comprehensive interventions should take at least 4 strategies into account: people-centered care strategies with context-specific and patient-sensitive manner, capacity building for qualified HCWs in PHC sectors, strengthening of cross-sector cooperation, and empowerment for TB patients’ self-management. For this, increased investment from governments for innovative and feasible measures such as internet and remote technologies and other new approaches is critical, especially in West China with insufficient resource. In addition, it is of great significance to summarize the beneficial practices of response to COVID-19 and integrate them into comprehensive interventions/strategies to promote TB control getting back on track of Ending TB [93]. And then implementation research is needed to develop strategies for adapting, implementing and enlarging those interventions in PHC sectors, particularly in West China with high TB burden and weak socio-economic condition.

## Conclusion

Since TB control in PHC sectors is important for ending TB in China, greater efforts on research about implementing TB control service program are of urgent needs to improve its outcomes. The treatment management of migrant TB patients and MDR-TB patients remains a huge challenge to TB control in Chinese PHC sectors under the integrated TB control model. In this review, we primarily conducted meta-analyses for outcome variables and obtained objective pooled results of existing studies. However, the number of included studies for each variable outcome was limited, and the heterogeneity was significant and was not effectively addressed through subgroup analyses with the same statistical methods and post hoc sensitivity analyses by excluding the low-quality studies, which indicated the need to be prudent to make conclusion. Therefore, it is deserved to urgently conduct more studies with more rigorous design to prove our findings and to analyze the status of delivering TB control service in PHC sectors, especially in West China with highest TB burden in China, in particular, Xinjiang, Tibet, Qinghai and Guizhou province where TB burden were ranked as the top four. Apart from currently reported factors mainly from TB patients and context of PHC sectors, more research evidences are needed to comprehensively identify factors associated with TB control service in PHC sectors. In conclusion, it is meaningful to design and implement comprehensive, community/village-based and patient-centered TB control interventions based on and suitable for regional characteristics and local medical resources as soon as possible.

### Electronic supplementary material

Below is the link to the electronic supplementary material.

Supplementary Material 1

Supplementary Material 2

Supplementary Material 3

Supplementary Material 4

Supplementary Material 5

## Data Availability

The datasets used during the current study are available from the corresponding author upon reasonable request.

## Abbreviations

AHRQ: Agency for Healthcare Research and Quality
BPHS: Basic Public Health Service
CDC: center for disease control and prevention
CHC: community health center
CHS: community health station
CNKI: China national knowledge infrastructure
DOT: Directly observed treatment
HCW: Healthcare workers
MDR-TB: Multi-drug resistant tuberculosis
NTP: National tuberculosis program
OR: Odds ratio
PHC: primary health care
PRISMA: Preferred reporting items for systematic reviews and meta-analyses
RR-TB: rifampicin-resistant tuberculosis
TB: Tuberculosis
TCM: TB case management
THC: township health center
VC: village clinic
