# Supplementary material for: Challenge of ending TB in China: tuberculosis control in primary healthcare sectors under integrated TB control model–a systematic review and meta-analysis
Source: BMC Public Health. 2024 Jan 11;24:163. doi: 10.1186/s12889-023-16292-5 (PMC10785344; doi:10.1186/s12889-023-16292-5)
Supplement: Supplementary file 2 — Supplementary Material 2 [file 12889_2023_16292_MOESM2_ESM.docx]

Additional file 2: Search strategies and results

1. **Electronic search strategy for PubMed**

| #1 | ((TB) OR tuberculosis) OR tuberculosis control |
| --- | --- |
|  |  |
|  |  |
| #2 | ((((primary health care sector) OR community health centre) OR community health service stations) OR township health centre) OR village clinic) |
|  |  |
|  |  |
| #3 | (China) OR Chinese |
| #4 | #1 And #2 And #3 |
| Results: 215 items | |

1. **Electronic search strategy for Cochrane**

| #1 | "TB" OR "tuberculosis" OR "tuberculosis control" |
| --- | --- |
|  |  |
|  |  |
| #2 | "primary health care sector" OR "community health centre" OR "community health service stations" OR "township health centre" OR "village clinic" |
|  |  |
|  |  |
|  |  |
|  |  |
| #3 | "China" OR "Chinese" |
| #4 | #1 and #2 and #3 |
| Results: 2 items | |

1. **Electronic search strategy for Web of Science**

| #1 | (TB OR tuberculosis OR tuberculosis control ) |
| --- | --- |
|  |  |
|  |  |
| #2 | (primary health care sector OR community health centre OR community health service stations OR township health centre OR village clinic) |
|  |  |
|  |  |
|  |  |
|  |  |
| #3 | ( China OR Chinese) |
| #4 | #1 AND #2 AND #3 |
| Results: 78 items | |

1. **Electronic search strategy for EMBASE**

| #1 | ("TB" OR "tuberculosis" OR "tuberculosis control") |
| --- | --- |
|  |  |
|  |  |
| #2 | ("primary health care sector" OR "community health centre" OR "community health service stations" OR "township health centre" OR "village clinic") |
|  |  |
|  |  |
|  |  |
|  |  |
| #3 | ("China" OR "Chinese") |
| #4 | #1 and #2 and #3 |
| Results: 2 items | |

1. **Electronic search strategy for CNKI**

| #1 | (SU = 'tuberculosis' OR SU = 'tuberculosis control') |
| --- | --- |
|  |  |
|  |  |
| #2 | (SU = 'primary' OR SU = 'primary health care sector' OR SU = 'primary medical institution' OR SU = 'community health centre' OR SU = 'community health service stations' OR SU= 'township health centre' OR SU = 'village clinic' ) |
|  |  |
|  |  |
|  |  |
|  |  |
| #3 | #1 and #2 |
| Results: 533 items | |

1. **Electronic search strategy for Wanfang**

| #1 | (Subject: (tuberculosis) or Subject: (tuberculosis control)) |
| --- | --- |
|  |  |
|  |  |
| #2 | (Subject: (primary) or Subject: (primary health care sector) or Subject: (primary medical institution) or Subject: (community health centre) or Subject: (community health service stations) or Subject: (township health centre) or Subject: (village clinic) ) |
|  |  |
|  |  |
|  |  |
|  |  |
| #3 | #1 * #2 |
| Results: 1351 items | |

1. **Electronic search strategy for VIP**

| #1 | (U=tuberculosis OR U=tuberculosis control) |
| --- | --- |
|  |  |
|  |  |
| #2 | (U=primary OR U=primary health care sector OR U=primary medical institution OR U=community health centre OR U=community health service stations OR U=township health centre OR U=village clinic) |
|  |  |
|  |  |
|  |  |
|  |  |
| #3 | #1 AND #2 |
| Results: 1862 items | |
