# Supplementary material for: Challenge of ending TB in China: tuberculosis control in primary healthcare sectors under integrated TB control model–a systematic review and meta-analysis
Source: BMC Public Health. 2024 Jan 11;24:163. doi: 10.1186/s12889-023-16292-5 (PMC10785344; doi:10.1186/s12889-023-16292-5)
Supplement: Supplementary file 3 — Supplementary Material 3 [file 12889_2023_16292_MOESM3_ESM.doc]

Additional file 3: Quality assessment of studies

**Table 1. Quality assessment criteria of cross-sectional studies**

| Item | Yes | No | Unclear |
| --- | --- | --- | --- |
| 1.Define the source of information (survey, record review) |  |  |  |
| 2.List inclusion and exclusion criteria for exposed and unexposed subjects (cases and controls) or refer to previous publications |  |  |  |
| 3.Indicate time period used for identifying patients |  |  |  |
| 4.Indicate whether or not subjects were consecutive if not population-based |  |  |  |
| 5.Indicate if evaluators of subjective components of study were masked to other aspects of the status of the participants |  |  |  |
| 6.Describe any assessments undertaken for quality assurance purposes (e.g., test/retest of primary outcome measurements) |  |  |  |
| 7.Explain any patient exclusions from analysis |  |  |  |
| 8.Describe how confounding was assessed and/or controlled |  |  |  |
| 9.If applicable, explain how missing data were handled in the analysis |  |  |  |
| 10.Summarize patient response rates and completeness of data collection |  |  |  |
| 11.Clarify what follow-up, if any, was expected and the percentage of patients for which incomplete data or follow-up was obtained |  |  |  |

**Table 2.** Quality assessment results of cross-sectional studies

| **Author** | **Year** | **Quality assessment criteria** | | | | | | | | | | | **Total Score** |
| --- | --- | --- | --- | --- | --- | --- | --- | --- | --- | --- | --- | --- | --- |
|  |  | Item 1 | Item 2 | Item 3 | Item 4 | Item 5 | Item 6 | Item 7 | Item 8 | Item 9 | Item10 | Item 11 |  |
| Jiang HX[34] | 2013 | 1 | 0 | 1 | 1 | 1 | 1 | 0 | 0 | 0 | 1 | 0 | 6 |
| Xu WX[35] | 2015 | 1 | 0 | 1 | 1 | 1 | 0 | 0 | 0 | 0 | 0 | 0 | 4 |
| Yang SY[36] | 2016 | 1 | 1 | 1 | 1 | 1 | 1 | 0 | 0 | 0 | 1 | 0 | 7 |
| Zheng YH[17] | 2016 | 1 | 0 | 1 | 1 | 1 | 0 | 0 | 0 | 0 | 0 | 0 | 4 |
| Zhong T[37] | 2016 | 1 | 1 | 1 | 1 | 1 | 1 | 0 | 0 | 0 | 1 | 0 | 7 |
| Liu T[38] | 2016 | 0 | 0 | 1 | 1 | 0 | 0 | 0 | 0 | 0 | 0 | 0 | 2 |
| Gao AH[15] | 2017 | 0 | 0 | 1 | 1 | 0 | 0 | 0 | 0 | 0 | 1 | 0 | 3 |
| Chen JH[39] | 2017 | 1 | 0 | 1 | 1 | 1 | 0 | 0 | 0 | 0 | 0 | 0 | 4 |
| Guo WR[41] | 2019 | 1 | 1 | 1 | 1 | 1 | 0 | 0 | 0 | 0 | 0 | 0 | 5 |
| Ou QY[42] | 2019 | 1 | 1 | 0 | 1 | 1 | 0 | 0 | 0 | 0 | 1 | 0 | 5 |
| Li WZ[16] | 2013 | 1 | 1 | 1 | 1 | 1 | 0 | 0 | 0 | 0 | 0 | 0 | 5 |
| Wu TY[45] | 2014 | 1 | 1 | 1 | 1 | 1 | 1 | 1 | 0 | 0 | 1 | 0 | 8 |
| Li Y[46] | 2014 | 1 | 1 | 1 | 1 | 1 | 1 | 1 | 1 | 1 | 1 | 0 | 10 |
| Chen W[48] | 2016 | 1 | 1 | 1 | 1 | 1 | 1 | 1 | 0 | 1 | 1 | 0 | 9 |
| Lin B[49] | 2017 | 1 | 0 | 1 | 1 | 0 | 0 | 0 | 0 | 0 | 1 | 0 | 4 |
| Mi YS[50] | 2018 | 1 | 1 | 1 | 1 | 1 | 1 | 0 | 0 | 0 | 1 | 0 | 7 |
| Zhang HW[19] | 2019 | 1 | 0 | 1 | 1 | 1 | 0 | 0 | 0 | 0 | 0 | 0 | 4 |
| Pu J[52] | 2019 | 1 | 1 | 1 | 1 | 1 | 1 | 1 | 0 | 1 | 1 | 0 | 9 |
| Ji XF[53] | 2020 | 1 | 0 | 1 | 1 | 0 | 0 | 0 | 0 | 0 | 0 | 0 | 3 |
| He YY[54] | 2020 | 1 | 0 | 1 | 1 | 0 | 1 | 0 | 0 | 0 | 0 | 0 | 4 |
| Xing W[55] | 2021 | 1 | 1 | 1 | 1 | 1 | 1 | 1 | 1 | 1 | 1 | 0 | 10 |
| Ou QY[56] | 2019 | 1 | 0 | 1 | 1 | 0 | 0 | 0 | 0 | 0 | 0 | 0 | 3 |
| Ming H[57] | 2019 | 1 | 0 | 1 | 1 | 0 | 0 | 0 | 0 | 0 | 1 | 0 | 4 |
| Wang JJ[65] | 2021 | 1 | 0 | 0 | 0 | 0 | 0 | 0 | 0 | 0 | 0 | 0 | 1 |
| Shi YY[66] | 2021 | 1 | 0 | 1 | 1 | 1 | 0 | 0 | 0 | 0 | 0 | 0 | 4 |

Notes:

1 indicates the answer of “Yes” in Item 1-4 and 6-11, and “No” or “Unclear” in Item5;

0 indicates the answer of “No” or “Unclear” in Item 1-4 and 6-11, and “Yes” in Item5.

**Table 3. Quality assessment results of cohort studies**

| **Author** | **Year** | **Quality assessment criteria** | | | | | | | | | **Total Score** |
| --- | --- | --- | --- | --- | --- | --- | --- | --- | --- | --- | --- |
|  |  | Exposed cohort truly representative | Non-exposed cohort drawn from the same community | Right method for ascertainment of exposure | Outcome of interest not present at start of study | Control of factors associated with treatment outcome or acceptability | Control of any additional factor (environmental factor or genes, demographic characteristics, treatment category, and diagnosis classification) | Quality of outcome assessment | Follow-up long enough for outcomes to occur | Complete accounting for cohorts |  |
| Yin J[40] | 2018 | 1 | 1 | 1 | 1 | - | 0 | - | 1 | 1 | 6 |
| Wang N[61] | 2019 | 1 | 1 | 1 | 1 | 1 | 1 | - | 1 | 1 | 8 |

Notes:

1 indicates the study met the criteria; 0 indicates the study did not meet the criteria; A dash indicates fulfillment of the criteria could not be determined.

**Table 4. Quality assessment results of before and after studies and non-randomized trials**

| **Author** | **Year** | **Type of study** | **Quality assessment criteria** | | | | | | | | | **Total Score** |
| --- | --- | --- | --- | --- | --- | --- | --- | --- | --- | --- | --- | --- |
|  |  |  | Is it clear in the study what is the ‘cause’ and what is the ‘effect’? | Were the participants included in any comparisons similar? | Were the participants included in any comparisons receiving similar treatment/care, other than  the exposure or intervention of interest? | Was there a control group? | Were there multiple measurements of the outcome both pre and post the intervention  /exposure? | Was follow up complete and if not, were differences between groups in terms of their follow up  adequately described and analyzed? | Were the outcomes of participants included in any comparisons measured in the same way? | Were outcomes measured in a reliable way? | Was appropriate statistical analysis used? |  |
| Ren PP[58] | 2015 | BAS | 1 | N/A | 1 | N/A | 1 | 1 | 1 | 0 | 1 | 6 |
| Yao CB[60] | 2016 | BAS | 1 | N/A | 1 | N/A | 0 | 1 | 1 | 0 | 0 | 4 |
| Zhou JH[70] | 2016 | BAS | 1 | N/A | 1 | N/A | 1 | 0 | 1 | 0 | 1 | 5 |
| Ye HM[18] | 2019 | IS(non-randomized) | 1 | 0 | 0 | 1 | 0 | 0 | 1 | 0 | 1 | 5 |
| Zhang LM[44] | 2021 | IS(non-randomized) | 1 | 1 | 0 | 1 | 1 | 0 | 1 | 0 | 1 | 6 |
| Liu XJ[14] | 2012 | IS(non-randomized) | 1 | 0 | 0 | 1 | 1 | 0 | 1 | 0 | 0 | 4 |
| Huang F[47] | 2016 | IS(non-randomized) | 1 | 0 | 0 | 1 | 1 | 1 | 1 | 1 | 1 | 7 |
| Li JH[51] | 2019 | IS(non-randomized) | 1 | 0 | 0 | 1 | 1 | 0 | 1 | 0 | 1 | 5 |
| Zhou QM[59] | 2015 | IS(non-randomized) | 1 | 0 | 0 | 1 | 1 | 0 | 1 | 0 | 1 | 5 |
| Li XF[63] | 2020 | IS(non-randomized) | 1 | 0 | 0 | 1 | 1 | 0 | 1 | 0 | 1 | 5 |

Notes:

1 indicates the answer of “Yes”;

0 indicates the answer of “No” or “Unclear”;

N/A indicates the answer of “Not applicable”

**Table 5. Quality assessment results of RCTs**

| **Author** | **Year** | **Quality assessment criteria** | | | |
| --- | --- | --- | --- | --- | --- |
|  |  | Allocation sequence  Generation (Adequate, inadequate, Unclear) | Allocation  Concealment (Adequate, inadequate, unclear) | Blinding (  assessors) (Adequate, inadequate) | Completeness of  follow up (Adequate, inadequate, unclear) |
| Lu QW[43] | 2021 | Adequate | Unclear | Inadequate | Adequate |
| Tang SP[62] | 2020 | Unclear | Unclear | Inadequate | Adequate |
| Yang WJ[64] | 2021 | Adequate | Unclear | Inadequate | Adequate |
| Huang MR[67] | 2021 | Unclear | Unclear | Inadequate | Adequate |
| Li XH[68] | 2018 | Unclear | Unclear | Inadequate | Adequate |
| Chen Y[69] | 2020 | Unclear | Unclear | Adequate | Adequate |

Notes:

Adequate indicates the study met the criteria;

Inadequate indicates the study did not meet the criteria;

Unclear indicates it is not mentioned
