# Supplementary material for: Challenge of ending TB in China: tuberculosis control in primary healthcare sectors under integrated TB control model–a systematic review and meta-analysis
Source: BMC Public Health. 2024 Jan 11;24:163. doi: 10.1186/s12889-023-16292-5 (PMC10785344; doi:10.1186/s12889-023-16292-5)
Supplement: Supplementary file 4 — Supplementary Material 4 [file 12889_2023_16292_MOESM4_ESM.docx]

Additional file 4: Results of assessment of publication bias


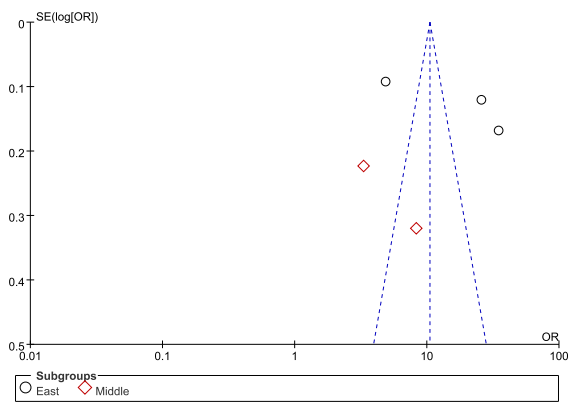


Figure 1 Assessment of publication bias of studies in arrival rate of tracing


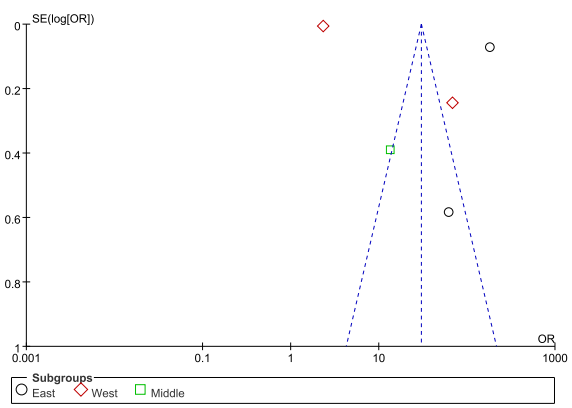


Figure 2 Assessment of publication bias of studies in arrival rate of referral


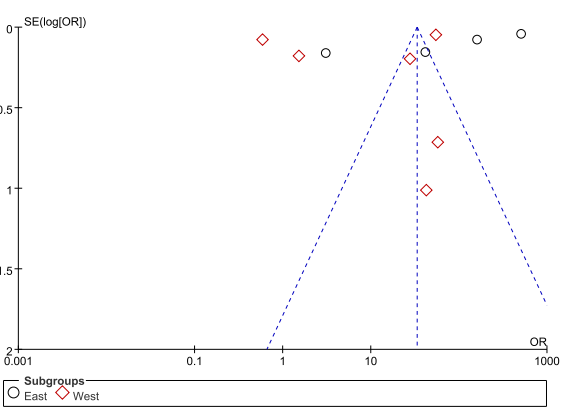


Figure 3 Assessment of publication bias of studies in TCM rate


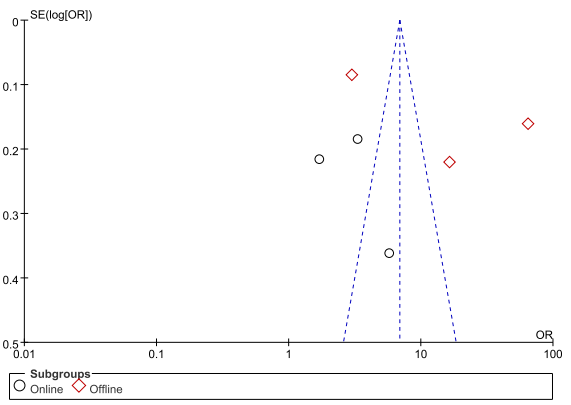


Figure 4 Assessment of publication bias of intervention studies in awareness of TB
