# Supplementary material for: Challenge of ending TB in China: tuberculosis control in primary healthcare sectors under integrated TB control model–a systematic review and meta-analysis
Source: BMC Public Health. 2024 Jan 11;24:163. doi: 10.1186/s12889-023-16292-5 (PMC10785344; doi:10.1186/s12889-023-16292-5)
Supplement: Supplementary file 5 — Supplementary Material 5 [file 12889_2023_16292_MOESM5_ESM.doc]

Additional file 5: Results of post hoc sensitivity analysis


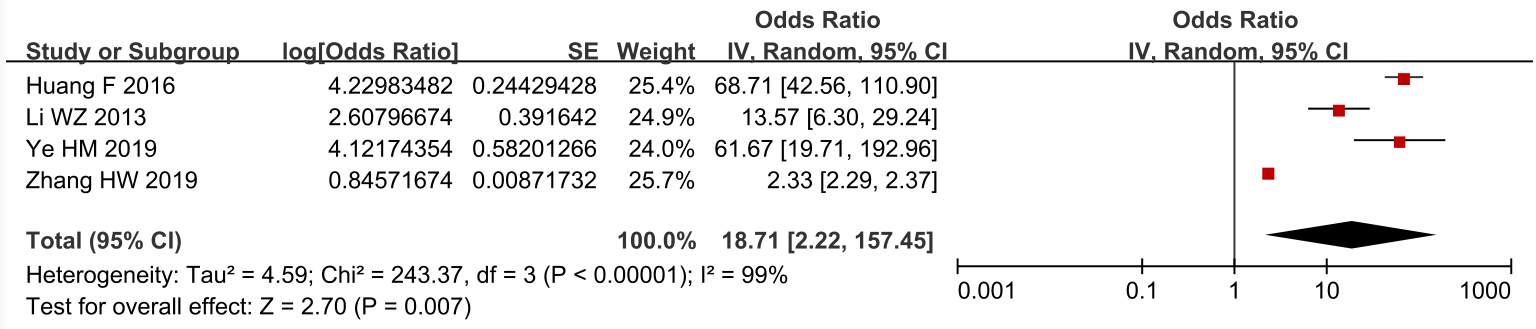


Figure 1 Post hoc sensitivity analysis for arrival rate of referral


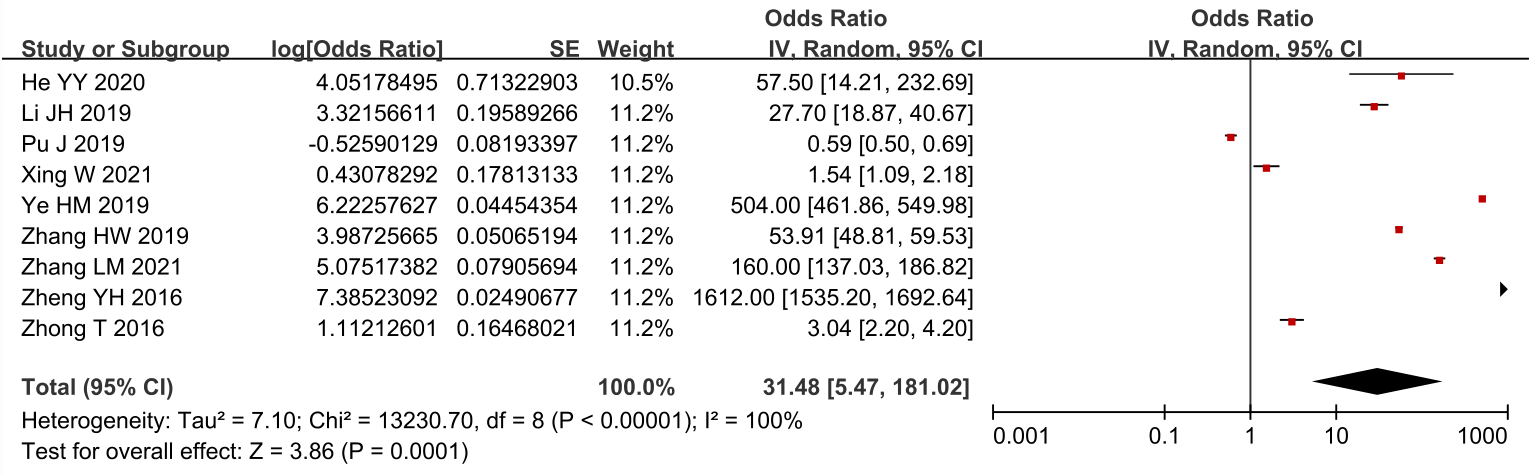


Figure 2 Post hoc sensitivity analysis for TCM rate


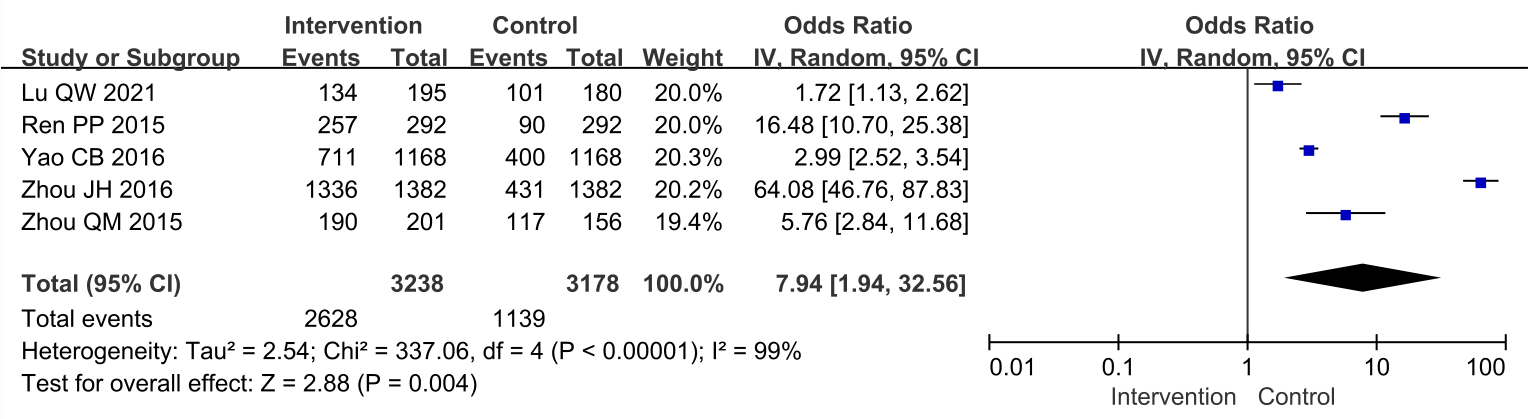


Figure 3 Post hoc sensitivity analysis for awareness of TB in intervention studies
